# Effects of exercise training in hypoxia versus normoxia on fat-reducing in overweight and/or obese adults: A systematic review and meta-analysis of randomized clinical trials

**DOI:** 10.3389/fphys.2022.940749

**Published:** 2022-08-23

**Authors:** Shuning Chen, Hao Su, Xinhong Liu, Qiaochu Li, Yingchi Yao, Jiachen Cai, Ye Gao, Qirui Ma, Yajun Shi

**Affiliations:** The School of Sports Science, Beijing Sport University, Beijing, China

**Keywords:** hypoxic exercise, normoxic exercise, obesity, body composition, glycometabolism, lipometabolism, meta-analysis

## Abstract

**Objective:** Fat loss theory under various oxygen conditions has been disputed, and relevant systematic review studies are limited. This study is a systematic review and meta-analysis to assess whether hypoxic exercise training (HET) leads to superior fat-reducing compared with normoxic exercise training (NET).

**Methods:** We searched PubMed, Web of Science, CNKI, ProQuest, Google Scholar, Cochrane Library, and EBSCOhost from inception to June 2022 for articles comparing the effects of hypoxic and normoxic exercise on body composition indicators, glycometabolism, and lipometabolism indicators in obese and overweight adults. Only randomized controlled trials (RCTs) were included. The effect sizes were expressed as standardized mean difference (SMD) and 95% confidence intervals (CI). Between-study heterogeneity was examined using the *I* ^*2*^ test and evaluated publication bias via Egger’s regression test. The risk of bias assessment was performed for each included trial using Cochrane Evaluation Tool second generation. The meta-analysis was performed by using R 4.1.3 and RevMan 5.3 analytic tools.

**Results:** A total of 19 RCTs with 444 subjects were analyzed according to the inclusion and exclusion criteria. Among them, there were 14 English literature and five Chinese literature. No significant difference in body composition (SMD -0.10, 95% CI -0.20 to -0.01), glycometabolism and lipid metabolism (SMD -0.01, 95% CI -0.13 to -0.10) has been observed when comparing the HET and NET groups. We only found low heterogeneity among trials assessing glycometabolism and lipometabolism (*I* ^*2*^ = 20%, *p =* 0.09), and no publication bias was detected.

**Conclusion:** The effects of HET and NET on fat loss in overweight or obese people are the same. The application and promotion of HET for fat reduction need further exploration.

## Introduction

Obesity has gradually become a global public health issue and a leading cause of death in most countries’ populations. According to the World Health Organization (WHO, 2020), the global prevalence of obesity had nearly tripled since 1975. Obesity is normally accompanied by the development of metabolic disorders such as insulin resistance, type II diabetes, and cardiovascular disease, which all have severe health effects for humans (Engin, 2017). In 2016, 39% of adults were overweight, and 13% were obese (WHO, 2020). As of 2017, nearly four million people die yearly due to obesity or overweight (WHO, 2020).

Obese people are more likely to have dyslipidemia (Gardner and Poehlman, 1995; Lu et al., 2008; Meyer et al., 2012). The type of dyslipidemia arising from the concerted action of obesity has been identified as “related metabolic dyslipidemia,” which is a clinical sign of elevated total cholesterol (TC), triglyceride (TG), low-density lipoprotein cholesterol (LDL-C), and reduced high-density lipoprotein cholesterol (HDL-C) in human blood (Su et al., 2021). Lipid metabolism reflects the metabolism of body fat and, to a certain extent, the risk of associated diseases (Lacour, 2001). Correction of dyslipidemia promotes body fat consumption and reduces cardiovascular risk in overweight and obese people (Kopin and Lowenstein, 2017; Vekic et al., 2019). Therefore, we need to focus on encouraging obese individuals to lose weight and correcting abnormalities in lipid metabolism.

Dietary intervention, exercise intervention, and medication intervention, among other things, are effective fat removal strategies (Hao et al., 2021). In terms of long-term fat reduction, exercise intervention outperforms diet and pharmaceutical interventions (Maillard et al., 2018). Traditional fat reduction workouts performed under normoxia efficiently lower extra body fat in obese patients. For example, moderate-intensity continuous training effectively boosts fat-burning capacity, improves body glycolipid levels, and enhances aerobic fitness (Rugbeer et al., 2021). High-intensity interval training can enhance aerobic capacity and insulin sensitivity, decrease blood glucose, promote adipose tissue breakdown, and improve vascular endothelial function (Batacan et al., 2017; Ryan et al., 2020). Sustained physical exercise decreases TC, TG, and LDL-C levels while increasing HDL-C levels (Burgomaster et al., 2008; Moholdt et al., 2012).

*Hypoxia* is a decrease in oxygen delivery to the body’s tissues caused by a fall in arterial blood oxygen saturation (Heinonen et al., 2016). Hypoxia and exercise synergistically affect lipid metabolism (Vogt et al., 2001). Hypoxia activates the peroxisome proliferator-activated receptor γ (PPARγ) and peroxisome proliferator-activated receptor -γ co-activator 1α (PGC1) (Zoll et al., 2003), which play an essential role in mediating the adaptive regulation of muscle fatty acid oxidation (Gilde and Van Bilsen, 2003). Similarly, active or passive hypoxia prevents obesity by activating the hypoxia-inducible factor (HIF) to maintain body mass and glucose homeostasis (Gaspar and Velloso, 2018). Reportedly, tissue hypoxia stimulates the production of HIF-1 (Vogt et al., 2001), improves glucose absorption and transport, enhances glycolysis, and accelerates lactate production, thereby increasing the efficiency of oxygen transport (Wenger, 2002) and ATP synthesis (Zoll et al., 2006). In addition, hypoxia increases blood serotonin levels in humans and rats (Urdampilleta et al., 2012). Serotonin regulates appetite, and rats show an anorexic response to serotonin administration (Urdampilleta et al., 2012). Long-term residents of high altitudes have appetite suppression and reduced body mass (Hill et al., 2011). Thus, a hypoxic environment may lead to a reduction in appetite in humans, which in turn may improve body mass loss.

Hypoxic exercise training (HET) is a method of exercise and fitness in a naturally occurring or artificially simulated plateau where the body is below normal oxygen conditions (Weng et al., 2006). An artificial hypoxic environment mainly includes a “hypoxic chamber” and a “hypoxic generator (Gürpinar Ö et al., 2018).” The hypoxic generator produces an absolute low partial pressure of oxygen by wearing a respiratory mask for the body to inhale hypoxia, resulting in a moderate hypoxic environment in the body (Hamlin et al., 2018). This hypoxic environment causes a series of anti-hypoxic physiological and biochemical reactions in the human body, affecting several physical signs (Wheelock et al., 2020). Studies have shown that exercising in hypoxic conditions helps alleviate a variety of cardiovascular, metabolic, and pulmonary illnesses, including obesity (Camacho-Cardenosa et al., 2018; Camacho-Cardenosa et al., 2019). Some studies claim that overweight or obese adults who perform HET experience more significant weight loss and greater improvements in body composition than those who perform normoxic exercise training (NET) (Kong et al., 2014; Park et al., 2019), whereas others found the opposite (Gatterer et al., 2015; Gonzalez-Muniesa et al., 2015). In another study (Netzer et al., 2008), the heart rate during exercise was same in the HET and NET groups (150 ± 5.3 beats/min vs.150 ± 4.6 beats/min), but the exercise load was significantly lower in the HET group than in the NET group (1.39 ± 0.20 w/kg vs.1.67 ± 0.15 w/kg, *p* < 0.001). At the same metabolic level, it is obvious that the subjects had to perform the exercise at a significantly lower intensity in hypoxic conditions than in normoxic conditions (Girard et al., 2017). In brief, hypoxia allows obese patients to achieve higher metabolic demands. At the same time, lower exercise intensity may also have a protective effect on the muscles/joints of obesity with orthopedic co-morbidities (Wiesner et al., 2010).

Previous literature reviews have critically examined the research potential of HET as an intervention for reducing body fat and cardiovascular health (Quintero et al., 2010; Kayser and Verges, 2013; Montero and Lundby, 2016; Girard et al., 2017; Hobbins et al., 2017; Camacho-Cardenosa et al., 2019; Dünnwald et al., 2019; Ramos-Campo et al., 2019; Kayser and Verges, 2021). Only five of them were systematic reviews. The research population for the systematic review by Montero and Lundby (2016) focused on the effects of exercise under hypoxic conditions on vascular health and was not overweight or obese. The systematic review by Hobbins et al. (2017) included both animal and human trials, and data from the included studies were not integrated and statistically analyzed to determine the intervention effects of hypoxic exercise on outcome indicators. The systematic review of (Camacho-Cardenosa et al. (2019) included only five studies and the conclusions obtained may be highly heterogeneous. The systematic review of Ramos-Campo et al. (2019) did not restrict the age of the subjects included in the study, and the age range may also induce bias into the study results. The systematic review by Dünnwald et al. (2019) studied the regular exercise population, but our review was conducted on a sedentary population. Accordingly, the purpose of this study was to systematically review and meta-analyze the differences in the effects of HET and NET on fat loss in overweight and obese individuals in order to determine whether exercise under hypoxic conditions can further contribute to fat loss and improved lipid metabolism levels.

## Methods

The review is reported according to the Preferred Reporting Items for Systematic reviews and Meta-Analyses (PRISMA) guidelines (Page et al., 2021).

### Search strategy

We performed a literature search using online databases such as PubMed, Web of Science, CNKI, ProQuest, Google Scholar, Cochrane Library, and EBSCOhost for relevant publications up to 9 June 2022. We used the standard Boolean operators (AND, OR) to concatenate search terms. The following combination of terms was used: “hypoxia” or “altitude” or “oxygen deficiency” or hypoxic exercise training” or “normoxia” or “normoxic exercise training.” The Boolean operator “AND” was used to combine these descriptors with “obesity” or “overweight,” “fat loss,” or “sedentary adults.” We also searched the aforementioned databases for systematic reviews and manually verified reference lists to identify studies that could have been omitted.

### Studies selection

The retrieved literature was imported into the reference management software EndNote. Duplicate publications were removed. The first round of preselection was based on the title and abstract. Those with incompatible topics were removed. The full text was downloaded and examined for the second preselection round to determine whether it met the inclusion criteria. Two investigators (SC and XL) independently conducted the database searches to find relevant publications. When conflicts occurred, a group discussion was held with a third investigator (QL).

### Inclusion criteria

Inclusion criteria followed the PICOS principles (i.e., population, intervention, comparison, outcome, and study design): 1) Participants were overweight or obese adults (18 years old), with criteria for diagnosing overweight (Body Mass Index >25 kg/m^2^) or/and obesity (all obesity categories; Body Mass Index >30 kg/m^2^) based on World Health Organization (WHO, 2020), and no physical restrictions or health conditions that would preclude evaluation and exercise intervention; 2) Studies that used a randomized controlled trial (RCT) design; 3) The original study article had to perform the exercise intervention under (normoxic or hypobaric) hypoxic condition; 4) The control group performed the same exercise as the intervention group under normoxic conditions (FiO_2_ = 0.21); 5) Outcome indicators were body mass (BM), body mass index (BMI), body fat percentage (BFR), fat body mass (FBM), waist circumference (WC), hip circumference (HC), waist-to-hip ratio (WHR), TC, TG, HDL-C, LDL-C, Glucose, and Insulin.

### Exclusion criteria

Studies that met these criteria were excluded: 1) Conference abstracts, dissertations, case reports, and animal experiments; 2) Participants who exercised frequently before the intervention; 3) Participants who were generally unable to exercise due to various diseases; 4) Inadequate data, with only a sub-set of data available before and after the intervention; 5) Repeatedly published studies; 6) Not English or Chinese studies.

### Data extraction

The extracted content primarily included the first author of the literature, the year of publication, the sample size, the gender and age of the subjects, the training details (intervention period, frequency, length, and intensity), the hypoxic condition, the type of hypoxia, and outcome indicators (Table 1). Information that was not accessible in the full text had partial data or was ambiguously contacted via email or other means to the authors, and if no answer was received, it was not included in the literature. If the standard deviation was not expressly stated in the text, it was calculated using the following formula (Higgins JPT et al., 2022):

$$\sqrt{{\text{SDpre}}^{2}+{\text{SDpost}}^{2}-2\times \text{Corr}\left(\text{pre},\text{post}\right)\times \text{SDpre}\times \text{SDpost}}$$

**TABLE 1:** Main characteristics of studies included in the meta-analysis.

Study	Participants characteristics					Intervention						
	Participants (M/F)		Age		BMI/BFR	Duration	Frequency	Type/modality	Exercise intensity	Hypoxic condition	Type of hypoxia	Outcome
	HET	NET	HET	NET								
Camacho-Cardenosa, A,2018	15 (0/15)	18 (0/18)	37.40 ± 10.25	40.05 ± 8.66	>25 kg/m^2^	12 weeks	3 days/wk	Repeated-sprint training; cycling	Exercise: (130% Wmax; 30 s); Active recovery: (55–65% Wmax; 3 min)	FiO_2_ = 17.2%	Normobaric	WC, HC, WHR, BFR,TG, TC, Glucose
Camacho-Cardenosa, A,2018^a^	13 (0/13)	15 (0/15)	44.43 ± 7.18	43.14 ± 7.67	>25 kg/m^2^	12 weeks	3 days/wk	IT, cycling	Exercise: (90% Wmax; 3 min); Active recovery: (55–65% Wmax; 3 min)	FiO_2_ = 17.2%	Normobaric	WC, HC, WHR, BFR,TG, TC, Glucose
Chacaroun, S,2020	12 (11/1)	11 (8/3)	52 ± 12	56 ± 11	> 27 kg/m^2^	8 weeks	3 days/wk	Endurance; cycling	75% (±2%) HRmax; 45 min	FiO_2_ = 13%	Normobaric	FBM, BMI, WC, HC, Glucose, Insulin
Fernandez, M,2018	12 (2/10)	11 (2/9)	34.8 ± 4.7	32.2 ± 8.4	>30 kg/m^2^	3 weeks	—	Endurance; walking	six different speeds; 60 min	FiO_2_ = 14.5%	Normobaric	BM, BMI,FBM,TC,TG,HDL-C,LDL-C, Glucose, Insulin
Gatterer, H,2015	16 (4/12)	16 (6/10)	50.3 ± 10.3	52.4 ± 7.9	>30 kg/m^2^	8 m	2 days/wk	Endurance; cycling	65–70% HRmax; 90 min	Exercise: FiO_2_ = 14.0 ± 0.2%; Rest: FiO_2_ = 12.2 ± 0.3%	Normobaric	BM, BMI, WC, HC, WHR, BFR,TG, TC, HDL-C, Glucose
Gonzalez-Muniesa, P,2015	14 (14/0)	12 (12/0)	25–50	25–50	>30 kg/m^2^	13 weeks	2 days/wk	Endurance/Strength; cycling	No mention; 1 h	FiO_2_ = 16.7%–13.7%	Normobaric	BM, BMI, WC, HC, WHR, BFR,TG, TC, LDL-C, HDL-C
Hobbins, L,2021	8 (4/4)	8 (5/3)	32.1 ± 10.2	41.1 ± 13.0	27–35 kg/m^2^	2 weeks	4 days/wk	IT; walking	self-paced,60min	FiO_2_ = 13.0%	Normobaric	BM, BMI
Jung, K,2020	12 (0/12)	10 (0/10)	47.2 ± 6.4	43.8 ± 8.6	>25 kg/m^2^	12 weeks	4∼5 days/wk	Endurance; Pilates	75% HRmax; 50 min	FiO_2_ = 14.5%	Normobaric	BM, BMI, BFR, TG, TC, LDL-C,HDL-C, Glucose, Insulin
Klug, L,2018	12 (12/0)	11 (11/0)	57.6 ± 2.2	55.0 ± 2.1	>305 kg/m^2^	6 weeks	3 days/wk	Endurance; running	50–60% HRmax; 60 min	FiO_2_ = 15%	Normobaric	BM, BMI, WC,HC,WHR,BFR,TG,HDL-C,LDL-C, Glucose, Insulin
Kong, Z,2014	10 (5/5)	8 (5/3)	19.8 ± 2.2	22.3 ± 1.7	>27.5 kg/m^2^	4 weeks	3∼4 days/wk	Endurance/Strength; cycling, running, stepping	60–70% HRmax; 1 h.40–50% maximal strength, three sets of 15–20 RM with 2–3 min of rest between sets	FiO_2_ = 16.4%–14.5%	Normobaric	BM, FBM, BMI, WHR
Kong, Z,2017	11	13	18–30	18–30	25.8 ± 2.3 kg/m^2^	5 weeks	4 days/wk	IT; cycling	8-s maximum; 60 repetitions	FiO_2_ = 15%	Normobaric	BM, BMI, FBM,BFR,TC,TG,HDL-C,LDL-C
Morishima, T,2014	9 (9/0)	11 (11/0)	30 ± 2	32 ± 3	>25 kg/m^2^	4 weeks	3 days/wk	Endurance; cycling	55%VO_2max_; 60 min	FiO_2_ = 15%	Normobaric	BM, FBM, BMI, BFR, TG, TC, LDL-C,HDL-C, Glucose, Insulin
Nishiwaki, M,2016	7 (0/7)	7 (0/7)	56 ± 1	56 ± 1	>24 kg/m^2^	8 weeks	4 days/wk	Endurance; aquatics	50% VO_2max_; 0.5 h	Simulation of 2000 m altitude (6001–6038 mmHg)	Normobaric	BM, BMI,BFR,Glucose
Park, H,2019	12 (12/0)	12 (12/0)	66.50 ± 0.90	66.50 ± 0.67	>25 kg/m^2^	12 weeks	3 days/wk	Endurance/Strength; cycling, running	60–70% HRmax; 90 min–100 min	FiO_2_ = 14.5%	Normobaric	BM, BFR, TG, TC, LDL-C,HDL-C
Wang, N,2012	11 (6/5)	7 (4/3)	19.5 ± 1.64	22.4 ± 2.07	H:34.62 ± 5.05 kg/m^2^ N:35.19 ± 5.07 kg/m^2^	4 weeks	6 days/wk	Endurance/Strength; cycling, running	M:100W, F:75 W; 4 h	FiO_2_ = 15.4%–14.8%	Normobaric	BM, FBM,BFR,WC,BMI,TC,TG,HDL-C,LDL-C
Wiesner, S,2010	24 (10/14)	21 (8/13)	42.2 ± 1.2	42.1 ± 1.7	H:33.1 ± 0.3 kg/m^2^ N:32.5 ± 0.8 kg/m^2^	4 weeks	3 days/wk	Endurance, running	65% VO_2max_,60 min	FiO_2_ = 15%	Normobaric	LDL-C, BFR, WC, Insulin
Yang, X,2014	10 (10/0)	8 (8/0)	22.50 ± 1.27	22.13 ± 2.17	>25 kg/m^2^	4 weeks	5 days/wk	Endurance; cycling, running	HR:140–150 beats/min; 1 h	FiO_2_ = 15.4%	Normobaric	BM, FBM, BMI, WHR, BFR, TG, TC, LDL-C,HDL-C
Zhang, N,2019	20 (20/0)	20 (20/0)	22.34 ± 2.15	21.87 ± 2.31	>25 kg/m^2^	1 m	5 days/wk	Endurance; cycling	65% V0_2max_; 60 min	FiO_2_ = 15%	Normobaric	BM, FBM, BMI, BFR, TG, TC, LDL-C, HDL-C
Zhao, S,2016	9	9	18.08 ± 1.79	18.24 ± 2.23	>30 kg/m^2^	8 weeks	5 days/wk	Endurance; cycling	65–75%V0_2max_	FiO_2_ = 14.7%	Normobaric	BM, BFR
Zheng, B,2020	10 (0/10)	10 (0/10)	19.60 ± 1.26	19.38 ± 0.74	>28%	6 weeks	3 days/wk	IT; cycling	Exercise: (64–76% HRmax; 10 min); Intervals: 3 min	FiO_2_ = 14.0 ± 0.2%	Normobaric	BM, FBM, BMI, BFR

Data are presented as mean or range. An article presented separate study groups distinguished by the presence or absence of a.

M, male; F, female; wk, weeks; d, day; FiO_2_, the fraction of inspired oxygen; IT, interval training; HR, heart rate; RM, repetition maximum; VO_2max_, maximal oxygen consumption; BM, body mass; BMI, body mass index; BFR, body fat rate; FBM, fat body mass; WC, waist circumference; HC, hip circumference; WHR, waist-to-hip ratio; TC, total cholesterol; TG, triglyceride; HDL-C, high-density lipoprotein cholesterol; LDL-C, low-density lipoprotein cholesterol.

SDpre is the pre-intervention SD, SDpost is the post-intervention SD, and Corr (pre, post) is the within-participant correlation coefficient, with the within-participant correlation set to 0.5 if no correlation is reported.

### Quality assessment

The included studies were assessed separately using the Revised Cochrane risk-of-bias tool for randomized trials (RoB 2) (Higgins JPT et al., 2022) in terms of the risk of bias from the randomization process, the impact of intervention assignment, the risk of bias due to missing outcome data, the risk of bias in outcome measures, the five major components, and the overall evaluation of the articles.

### Data synthesis and statistical analysis

1) The collected trials were meta-analyzed using R (RStudio V4.13, Boston, MA, United States) and RevMan (RevMan 5.3, Cochrane Collaboration, Oxford, United Kingdom) analytic tools, and all experimental data were continuous variables. The units of measurement for the outcome indicators were different, so standardized mean difference (SMD) effect scales and 95% confidence interval (CI) were utilized for statistical purposes.
2) *I* ^*2*^ was used to assess the heterogeneity of a study. When *I* ^*2*^ = 0, there was no heterogeneity among the included trials, and a fixed-effects model was used for analysis. When *I* ^*2*^ > 40%, there was a significant probability of heterogeneity, and a random-effects model was used for analysis, along with a sensitivity analysis to eliminate the test for high heterogeneity. When the heterogeneity was too tremendous and determining the cause was problematic, descriptive analysis was performed.
3) Assessment of potential modifiers (the age of participants, the duration of the exercise intervention, the frequency of the intervention, and the level of hypoxia) of body composition indicators, glycometabolism, and lipometabolism indicators through subgroup analysis. Sensitivity analysis was done to investigate the sources of heterogeneity and assess the results’ stability by removing each test individually.
4) We evaluated publication bias using Egger’s regression test (Egger et al., 1997). When the sample size of the included literature was less than 15, publication bias testing was not necessary because of the small sample size and low test power (Cumpston et al., 2019).
5) If *p*< 0.05, it is considered a significant difference.

## Results

### Study selection and characteristics

**FIGURE 1:**
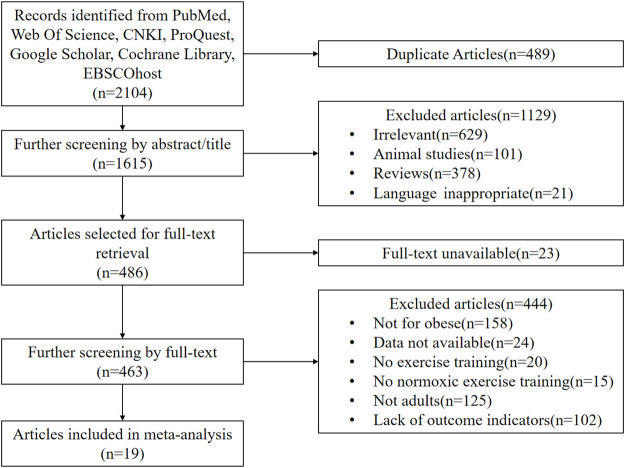
Flow diagram of the process of article selection.

The original literature search yielded 2,104 studies, of which 2082 were written in English and 22 were written in Chinese. After deleting duplicates with Endnote software and reviewing the titles and abstracts of the literature for initial screening, 486 studies were collected, of which 23 were discarded due to the lack of full text. Four hundred forty-four were excluded after reading the full text of the remaining 463 studies, and 19 RCTs were finally included, including 14 English and five Chinese studies (Wiesner et al., 2010; Wang et al., 2012; Kong et al., 2014; Morishima et al., 2014; Yang et al., 2014; Gatterer et al., 2015; Gonzalez-Muniesa et al., 2015; Nishiwaki et al., 2016; Zhao and Shi, 2016; Kong et al., 2017; Camacho-Cardenosa et al., 2018; Fernández Menéndez et al., 2018; Klug et al., 2018; Park et al., 2019; Zhang, 2019; Chacaroun et al., 2020; Jung et al., 2020; Zheng et al., 2020; Hobbins et al., 2021). Figure 1 depicts the procedure for incorporating the literature.

Table 1 contains the essential information on 19 studies, one of which incorporated two types of HET (Camacho-Cardenosa et al., 2018); hence the study was included twice. A total of 444 subjects were included in the study, all of whom were overweight or obese adults (aged 18–66 years) without metabolic diseases such as heart disease or type II diabetes. Six studies contained only male subjects, four studies contained only female subjects, seven studies contained both male and female subjects, and two studies did not specify the gender of the subjects in the text. The exercise mode was identical between the HET and NET groups, with 11 studies involving continuous aerobic exercise, primarily jogging, cycling, and aerobic exercise, with an average duration of more than 20 min per exercise session; four studies involving intermittent exercise, with three studies involving high-intensity interval exercise, and one study involving moderate-intensity interval exercise. The four studies used a combination of aerobic and strength training. The FiO_2_ of the HET group ranged from 13% to 17.2%, whereas the NET group was exposed to normal atmospheric conditions (FiO_2_ = 21%).

### Risk-of-bias assessment

**FIGURE 2:**
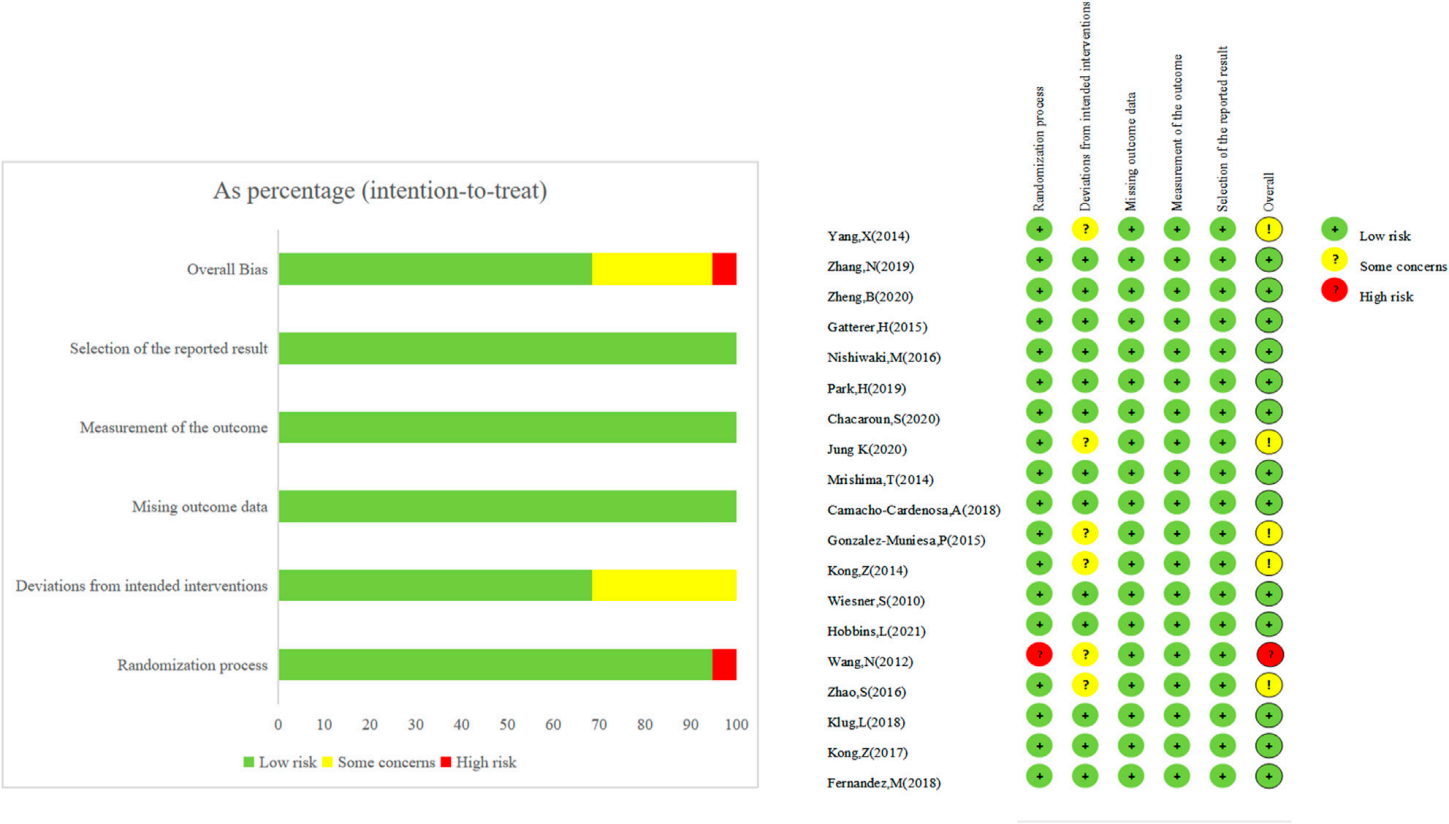
The risk assessment of bias.

Among the 19 included studies, 18 were considered bias-free overall, while one was determined to be high-risk overall because randomization grouping was not specified in the text. Figure 2 provides further details. Regarding blinded assessment, one study utilized the double-blinded approach, six used the single-blinded method, while the remaining 12 did not accomplish experimental implementer and participant blinded evaluation. The remaining 12 research was not blinded to participants or personal information, and the outcome indicators of six of these studies may have been impacted due to the absence of blinding, putting these six studies at risk. In contrast, the remaining 13 studies were at low risk.

### Publication bias

**FIGURE 3:**
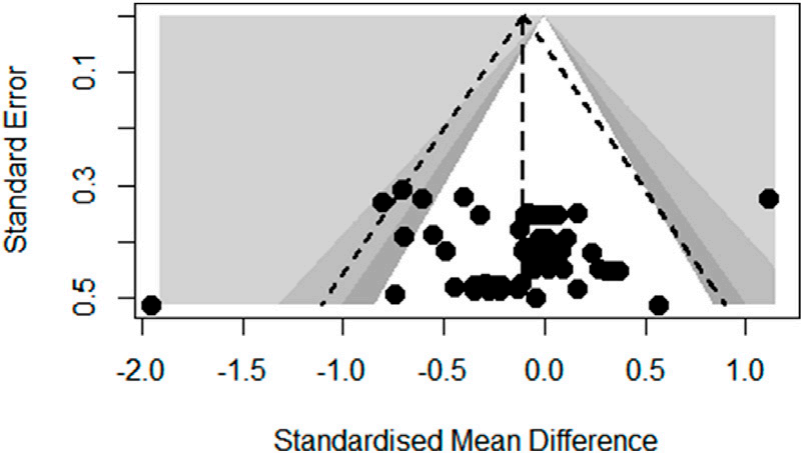
Funnel plot of body composition outcomes.

**FIGURE 4:**
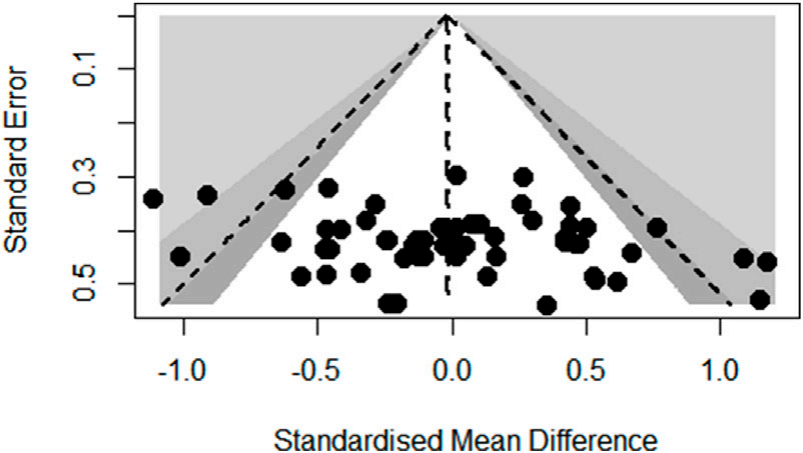
Funnel plot of glycometabolism and lipometabolism outcomes.

Funnel plot analysis of body composition outcomes showed slight asymmetry but Egger’s regression test did not reach statistical significance (*p* = 0.6672; see Figure 3). Also, the funnel plot of glycometabolism and lipometabolism outcomes showed reasonable symmetry and Egger’s test did not reach statistical significance (*p* = 0.9647; see Figure 4).

### Meta-analysis

**FIGURE 5:**
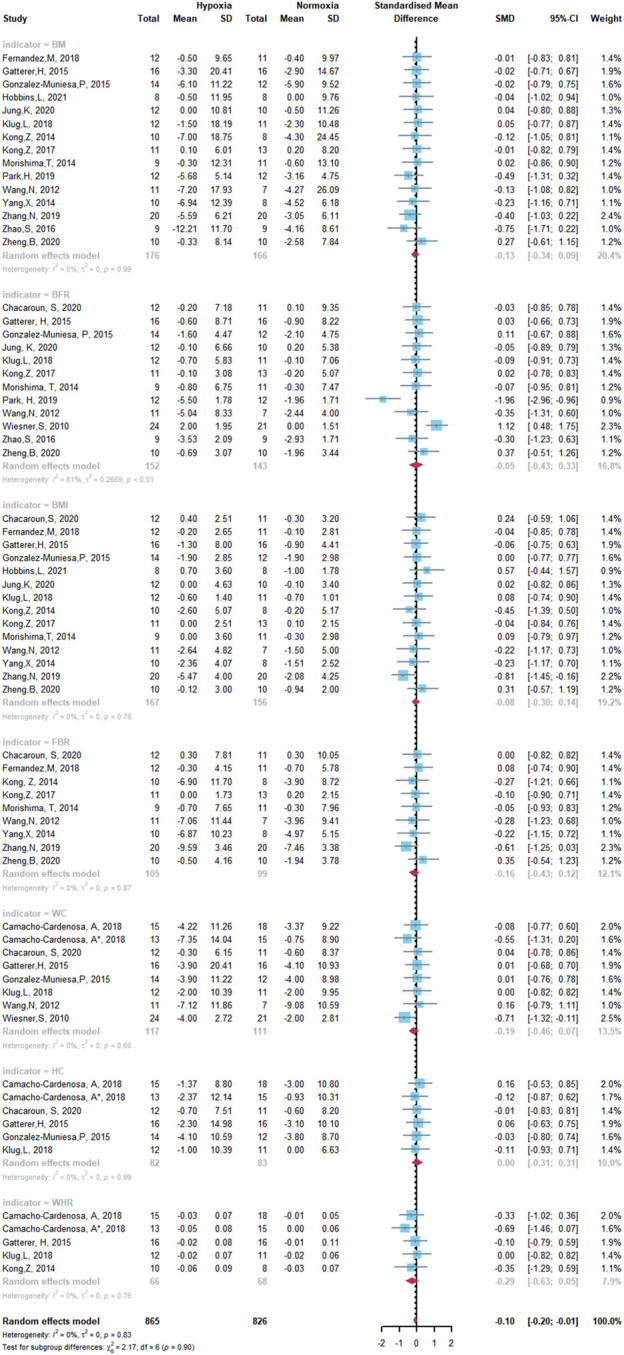
Forest Plot of the Meta-analysis on body composition.

**FIGURE 6:**
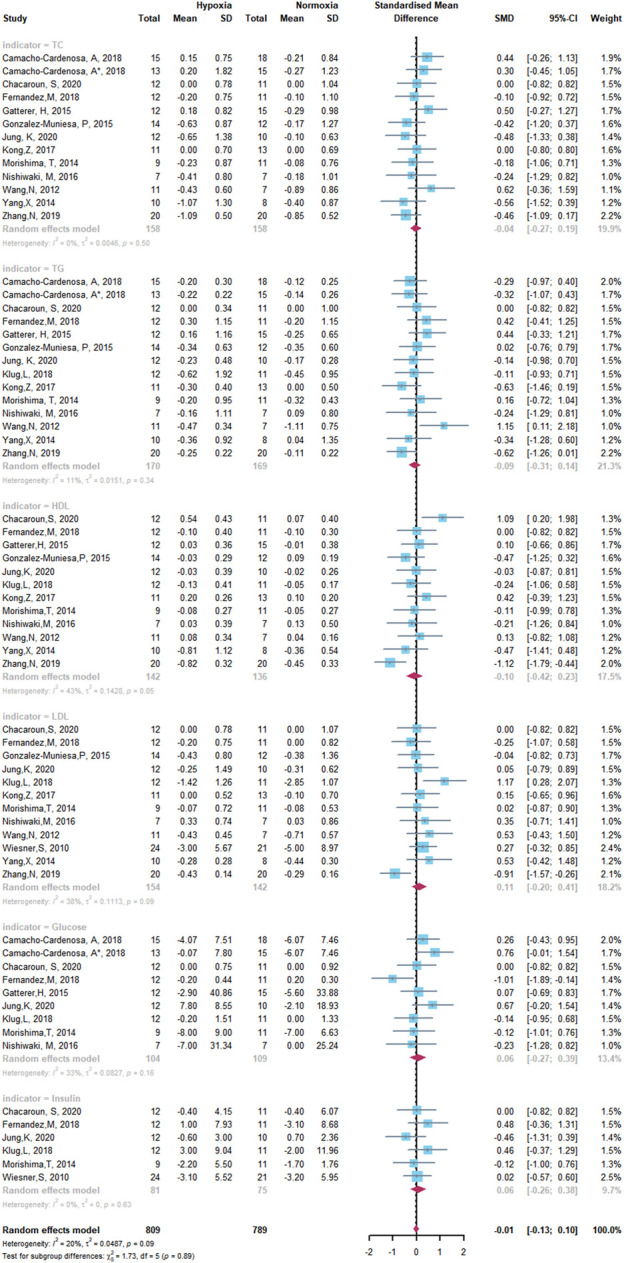
Forest Plot of the Meta-analysis on glycometabolism and lipometabolism.

The results of the meta-analysis of all studies are shown as forest plots in Figure 5 and Figure 6. A random effects model was considered the main method for meta-analysis to account for the heterogeneity between studies.

The overall meta-analysis of the effects of HET and NET on body composition comprised 69 SMDs from 19 trials (Figure 5). There was no significant difference in the improvement of body composition with HET and NET and no heterogeneity among the studies (SMD -0.10, 95% CI -0.20 to -0.01; *p =* 0.90, *I* ^2^ = 0%). Similarly, for studies conducted on glycometabolism and lipometabolism, our meta-analysis reveals no difference between HET and NET and low heterogeneity among the studies (SMD -0.01, 95% CI -0.13 to -0.10; *p =* 0.89, *I* ^2^ = 20%) (Figure 6).

### Subgroup analyses

The effects of HET and NET on the body composition, glycometabolism, and lipometabolism of overweight and obese adults may be influenced by the age of participants, intervention duration, frequency, and level of hypoxia. Subgroup analysis demonstrated that the age of participants, intervention duration, frequency, and level of hypoxia did not change the results of the meta-analysis (Table 2).

**TABLE 2:** Subgroup analyses of the effects of exercise training performed in hypoxia versus normoxia on reducing the fat.

Subgroup	Frequency (d/wk)						Duration (wk)						FiO_2_ (%)						age (yr)					
		n	SMD [95%CI]	*p* values of SMD	*I* ^*2*^	*p* values of I^2^		n	SMD [95%CI]	*p* values of SMD	*I* ^*2*^	*p* values of I^2^		n	SMD [95%CI]	*p* values of SMD	*I* ^*2*^	*p* values of I^2^		n	SMD [95%CI]	*p* values of SMD	*I* ^*2*^	*p* values of I^2^
BM	<4	6	0.03 [−0.31, 0.36]	0.88	0%	0.99	<8	9	−0.03 [−0.32, 0.26]	0.84	0%	1	<15	6	−0.06 [−0.40, 0.29]	0.74	0%	0.77	<45	10	−0.14 [−0.41, 0.13]	0.33	0%	0.96
	≥4	7	−0.19 [−0.56, 0.18]	0.31	0%	0.84	≥8	5	−0.13 [−0.53, 0.27]	0.52	0%	0.59	≥15	5	0.02 [−0.46, 0.50]	0.94	0%	0.99	≥45	3	−0.14 [−0.58, 0.30]	0.54	0%	0.06
BMI	<4	7	0.03 [−0.28, 0.34]	0.83	0%	0.94	<8	9	−0.09 [−0.40, 0.23]	0.58	0%	0.71	<15	5	0.13 [−0.21, 0.47]	0.44	0%	0.92	<45	9	−0.10 [−0.38, 0.18]	0.48	0%	0.71
	≥4	6	−0.09 [−0.49, 0.32]	0.67	0%	0.44	≥8	4	0.08 [−0.27, 0.44]	0.65	0%	0.96	≥15	5	−0.12 [−0.55, 0.31]	0.58	0%	0.52	≥45	3	0.07 [−0.38, 0.51]	0.77	0%	0.86
FBM	<4	4	−0.10 [−0.44, 0.23]	0.55	0%	0.87	<8	3	−0.10 [−0.44, 0.23]	0.55	0%	0.87	<15	2	0.13 [−0.35, 0.62]	0.60	0%	0.84	<45	8	-	-	-	-
	≥4	5	−0.31 [−0.93, 0.31]	0.33	0%	0.44	≥8	5	−0.01 [−0.40, 0.38]	0.97	0%	0.87	≥15	5	−0.22 [−0.73, 0.29]	0.39	0%	0.67	≥45	1	-	-	-	-
BFR	<4	5	0.29 [−0.12, 0.70]	0.16	41%	0.13	<8	4	0.22 [−0.26, 0.70]	0.37	51%	0.07	<15	4	0.02 [−0.39, 0.43]	0.91	0%	0.78	<45	6	0.19 [−0.32, 0.69]	0.46	54%	0.05
	≥4	3	−0.22[−0.74, 0.30]	0.41	0%	0.88	≥8	4	−0.03 [−0.42, 0.37]	0.90	0%	0.92	≥15	3	0.38 [−0.00, 0.76]	0.05	63%	0.04	≥45	5	−0.46 [−1.29, 0.36]	0.27	75%	0.007
TC	<4	6	0.14 [−0.18, 0.46]	0.39	0%	0.51	<8	3	0.05 [-0.38, 0.48]	0.82	0%	0.64	<15	3	0.00 [−0.40, 0.41]	0.98	0%	0.41	<45	8	−0.01 [−0.28, 0.27]	0.97	0%	0.40
	≥4	5	−0.14 [−0.55, 0.27]	0.43	0%	0.50	≥8	7	0.01 [−0.28, 0.30]	0.95	8%	0.37	≥15	5	0.18 [−0.20, 0.57]	0.36	0%	0.69	≥45	3	0.15 [−0.34, 0.65]	0.54	0%	0.49
TG	<4	5	−0.03 [−0.33, 0.26]	0.84	0%	0.83	<8	3	0.07 [−0.40, 0.54]	0.76	41%	0.13	<15	4	0.19 [−0.22, 0.60]	0.36	0%	0.68	<45	8	−0.13 [−0.50, 0.25]	0.51	42%	0.10
	≥4	5	−0.08 [−0.65, 0.49]	0.78	46%	0.11	≥8	7	−0.08 [−0.38, 0.22]	0.62	0%	0.85	≥15	5	−0.33 [−0.71, 0.05]	0.09	0%	0.85	≥45	4	0.06 [−0.36, 0.49]	0.76	0%	0.70
HDL	<4	3	0.04 [−0.33, 0.41]	0.83	48%	0.11	<8	3	−0.04 [−0.43, 0.36]	0.86	0%	0.66	<15	4	0.37 [−0.30, 1.03]	0.28	49%	0.14	<45	6	−0.32 [−0.69, 0.05]	0.09	59%	0.05
	≥4	5	0.01 [−0.40, 0.41]	0.98	0%	0.70	≥8	5	0.08 [−0.30, 0.46]	0.73	45%	0.12	≥15	3	0.03 [−0.45, 0.52]	0.89	0%	0.50	≥45	4	0.19 [−0.24, 0.62]	0.39	41%	0.13
LDL	<4	3	0.18 [−0.23, 0.60]	0.39	30%	0.22	<8	4	0.28 [−0.12, 0.68]	0.18	30%	0.21	<15	5	−0.07 [−0.54, 0.41]	0.78	0%	0.87	<45	7	−0.08 [−0.52, 0.37]	0.73	48%	0.09
	≥4	5	0.34 [−0.13, 0.82]	0.15	0%	0.86	≥8	4	0.06 [-0.37, 0.48]	0.26	0%	0.94	≥15	4	0.54 [−0.61, 1.68]	0.36	78%	0.03	≥45	4	0.50 [−0.22, 1.22]	0.17	46%	0.16
Glucose	<4	6	0.16 [−0.16, 0.48]	0.32	0%	0.62	<8	3	−0.41 [−0.91, 0.08]	0.10	25%	0.26	<15	4	−0.05 [−0.46, 0.36]	0.81	59%	0.06	<45	4	0.06 [−0.34, 0.45]	0.78	68%	0.02
	≥4	2	0.31 [−0.36, 0.98]	0.37	40%	0.20	≥8	6	0.29 [−0.04, 0.61]	0.09	0%	0.56	≥15	5	0.17 [−0.20, 0.54]	0.37	0%	0.43	≥45	4	−0.05 [−0.47, 0.37]	0.81	0%	0.97
Insulin	<4	4	-	-	-	-	<8	4	0.18 [−0.20, 0.55]	0.36	0%	0.64	<15	3	0.01 [−0.47, 0.49]	0.96	15%	0.31	<45	3	0.10 [−0.32, 0.52]	0.63	0%	0.57
	≥4	1	-	-	-	-	≥8	2	−0.22 [−0.81, 0.37]	0.47	0%	0.45	≥15	3	0.10 [−0.32, 0.52]	0.65	0%	0.60	≥45	2	0.23 [−0.36, 0.81]	0.45	0%	0.44

n, number of studies. -, absence of studies for a given subgroup. BM, body mass; BMI, body mass index; BFR, body fat rate; FBM, fat body mass; WC, waist circumference; HC, hip circumference; WHR, waist-to-hip ratio; TC, total cholesterol; TG, triglyceride; HDL-C, high-density lipoprotein cholesterol; LDL-C, low-density lipoprotein cholesterol.

## Discussion

This review aimed to examine the effect of hypoxia on fat loss compared to equivalent training in normoxia in overweight or obese adults. The key finding of this meta-analysis was that training in hypoxia elicited comparable responses in body composition, glycometabolism, and lipometabolism in individuals. In addition, we found that the subgroup analysis of different training duration, training frequency, hypoxia levels, and the age of participants not significantly improved body composition, glycometabolism, and lipometabolism values. These findings may have important implications for obese patients and multidisciplinary teams involved in obesity management.

### Hypoxic exercise training and normoxic exercise training effects on body composition in obese and overweight people

BM, BMI, FBM, and BFR were reliable and valid for identifying adults at increased risk for death and morbidity related to overweight and obesity (Aronne, 2002). This review showed no significant positive effect on body composition for both hypoxic and normoxic conditioning. This is an important finding because it has previously been reported that active hypoxia may reduce BM in animals and humans (Hobbins et al., 2017). Early studies have found that hypoxia exposure at high altitudes can cause weight loss and changes in other body composition (Ge et al., 2010). A meta-analysis of studies by Dünnwald et al. (2019) showed that greatest reductions in BM and FBM were observed in moderate altitudes (1,500–3,500 m) when exposure was active and the duration of hypoxia exposure was more than 42 days (6 weeks). Active exposure primarily consisted of hiking, trekking, and sometimes swimming (e.g., activity during moderate altitude vacations) (Dünnwald et al., 2019). The studies included in this review simulated oxygen concentration conditions at moderate altitudes (FiO_2_: 13.5%–17.4%), and the active exposure consisted of running and cycling in the hypoxic chamber. It follows that subjects in the studies included in this review were exposed to hypoxic environments for less hours per day than those included in the studies by Dünnwald (Dünnwald et al., 2019). Thus, the insufficient daily hypoxic exposure time may have contributed to the insignificant difference between HET and NET in terms of fat loss.

Another factor contributing to weight loss in hypoxic conditions was decreased caloric intake through meals as a result of diminished appetite (Westerterp-Plantenga et al., 1999). Highlanders’ negative energy balance was the primary cause of weight loss (Hamad and Travis, 2006). According to animal studies, 21-day-old rats exposed to a low-pressure chamber at a simulated altitude of 6100 m only had 54.6% of the food intake and 32.7% of the body weight gain of normoxic rats (Bozzini et al., 1987). Energy intake was also reduced in humans exposed to artificial hypoxia (De Glisezinski et al., 1999). Due to the short duration of hypoxia exposure per day in the studies included in this review, subjects may not have been affected by reduced appetite.

More studies have also found non-significant improvements in body composition in the hypoxic condition. For instance, Wiesner et al. (Wiesner et al., 2010) discovered that in a 4-weeks HET trial of normal-weight people, HET was not considerably more successful in lowering BM compared to NET. Netzer et al. (Netzer et al., 2008)reported an insignificant reduction in BM and BMI in the hypoxic group. Similarly, in another study, subjects exercising in a hypoxic (FiO_2_ = 15.0%) and normoxic environment (moderate intensity cycling, three times per week for 4 weeks) did not show significant changes in BMI and FBM. However, the normoxic group demonstrated a slight decrease in BM following the intervention compared to the hypoxic group (-1% and -0.5%, respectively) (Morishima et al., 2014). Subgroup analysis of this review showed comparable effects of HET and NET on body composition at less than 8 weeks. Therefore, hypoxia may take time to have a physiological effect on the body. Nevertheless, the longer duration of the intervention (8 weeks–8 months) did not result in significant differences between the HET and NET groups either. This could have led to subjects already having adapted to the hypoxic environment. When people have been exposed to hypoxia for long periods of time, the adaptation of body composition may rapidly plateau and, without periodic adjustment, may not continue the benefits of hypoxia to the organism (Hobbins et al., 2017).

In addition to BM, BMI, FBM, and BFR, the risk of overweight and obesity was strongly associated with excess abdominal fat and lower fitness level. The results of two previous studies (Wiesner et al., 2010; Shin et al., 2018) showed a significant decrease in WC in subjects after HET (aerobic training, 60 min/d, VO_2max_ of 65%, and FiO_2_ of 14.5–15%), but no significant changes in NET. Another study (Camacho-Cardenosa et al., 2018) also found a significant reduction in WC and WHR after 12 weeks of HET. These studies suggested that HET positively affected abdominal fat reduction, which may be related to the enhanced oxidation of lipids after exercise (Camacho-Cardenosa et al., 2018). It is noteworthy that reducing abdominal fat requires adequate exercise duration and intensity. According to the American College of Sports Medicine (Wilkins, 2006), people who are overweight or obese should achieve the following recommended program of moderate-intensity exercise for 50–60 min/day and 300 min/week when exercising for weight loss. Only four trials included in this review had exercise protocols that met the exercise regimen recommended by the American College of Sports Medicine (Wang et al., 2012; Yang et al., 2014; Zhao and Shi, 2016; Zhang, 2019). In addition, when people exercised in a hypoxic environment as opposed to a normoxic one, there was a tremendous increase in heart rate, more production of blood lactate, and more significant subjective fatigue (Bouissou et al., 1987). It follows that exercise in a hypoxic environment may be more “challenging”, especially for obese individuals. In other words, exercising in an environment with low levels of hypoxia may cause physiological discomfort, leading to reduction in total workload. Hence, more data from randomized trials are required to determine the optimal intervention duration, intervention frequency, and hypoxia level for fat loss in NETs.

### Hypoxic exercise training and normoxic exercise training effects on glycometabolism and lipometabolism in obese and overweight people

The current meta-analysis showed that HET and NET did not significantly improve lipid metabolism, which is generally consistent with previous studies (Netzer et al., 2008; Wiesner et al., 2010). Differences in the current findings may be primarily related to the total duration of the training. It has been shown that subjects with NET have significantly increased HDL-C levels (Costa et al., 2019). Netzer (Netzer et al., 2017) found that subjects who completed 8 weeks of exercise under hypoxia or normoxia showed a greater increase in TG, TC, and HDL-C. In other studies of similar exercise intensity, TG, TC, and HDL-C did not change after completing 4 weeks of exercise training in the HET and NET groups (Wiesner et al., 2010; Morishima et al., 2014). Interestingly, all intervention groups of these findings completed the same type of exercise at an ‘absolute’ intensity, i.e., an intensity regardless of the environmental condition. The proportion of carbohydrate oxidation increase under hypoxia condition compared to the normoxic condition (Jung et al., 2020). However, exercising in a hypoxic environment puts extra stress on the metabolism of lipids, which the body must overcome to oxidize further substances that provide energy in the muscles (Kelly and Basset, 2017; Jung et al., 2020). As mentioned above, the hypoxic environment makes exercising more “harder.” Therefore, we suggest that the intensity of exercise achieved by people exercising in a normoxic environment may be what stimulates lipid metabolism.

Previous studies have shown HET increases the relative glucose oxidation rates of subjects (Geiser et al., 2001; Vogt et al., 2001; Hoppeler et al., 2003). This phenomenon was attributed to the trans-activation of HIF-1 (Wiesner et al., 2010). In this review, exercise training under hypoxic and normoxic conditions elicited similar responses in obese patients, suggesting that HET had no additional effect on fasting insulin and glucose metabolism in obese subjects.

It is well-established the age-related variations in glycometabolism and lipometabolism (Tessari, 2000). Nevertheless, subgroup analysis of this review demonstrated that neither youth (18–44 years) nor middle-aged subjects (45–66 years) exhibited changes in lipid metabolism between HET and NET. Only five trials in this review had subjects over 45 years of age (Nishiwaki et al., 2016; Klug et al., 2018; Park et al., 2019; Chacaroun et al., 2020), which may lead to biased results. Furthermore, most trials included in this review (Wiesner et al., 2010; Yang et al., 2014; Gatterer et al., 2015; Nishiwaki et al., 2016; Kong et al., 2017; Camacho-Cardenosa et al., 2018; Fernández Menéndez et al., 2018; Klug et al., 2018; Park et al., 2019; Zhang, 2019; Chacaroun et al., 2020; Jung et al., 2020; Zheng et al., 2020; Hobbins et al., 2021) did not investigate dietary intake and daily activity in the HET and NET groups during the hypoxic exercise intervention. As a result, we cannot rule out the effect of the age of participants, food intake, and daily activities on glycometabolism and lipometabolism.

There were several limitations to this review that should be taken into account. Even though a systematic literature search was conducted, it was possible that some relevant studies were not included in this meta-analysis if our search algorithm did not capture them. In addition, there was unavoidable variation in the design protocols of different literature, such as individual subject differences, compliance, intervention duration, and exercise format. These uncontrollable variables may have contributed to the heterogeneity in this study.

To conclude, this systematic review with meta-analysis showed that HET and NET have similar effects on fat loss in overweight and obese adults. Furthermore, subgroup analysis also revealed that hypoxia had no effect on body composition, glycometabolism, and lipometabolism when potential parameters (i.e., the age of participants, hypoxia dose, exercise frequency, and duration) are altered. Thus, application and promotion of HET in fat reduction require additional exploration. However, due to some limitations, more RCTs with larger sample sizes are needed to further understand exercise’s effectiveness on fat loss under different oxygen conditions.

## Data Availability

The original contributions presented in the study are included in the article/Supplementary Material, further inquiries can be directed to the corresponding author.
